# 

**Published:** 1896-05-30

**Authors:** 


**Notice to Advertisers and Subscribers.**

All Advertisements, Orders for Copies of The Hospital and Business Communications should be addressed to **THE MANAGER, (Not to The Editor),** The Hospital, 428, Strand, London, W.C.

The Cost of Subscription, post free, is as follows:—Per week, 2½d.; per quarter, 2s. 8d.; per half-year, 5s. 3d.; per annum, 10s. 6d. Foreign:—Per Annum, 15s. 2d.; payable, in all cases, in advance.

### SCRAPS AND GLEANINGS.

It is proposed to establish a cottage hospital at Crawley, Sussex.

The Hastings Provident Dispensary was wound up at the annual meeting held on the 30th ult.

It has been estimated that throughout the world 67 persons die and 70 are born every minute of the day.

An extension of the Middlesbrough Sanatorium is under consideration in order to provide accommodation for small-pox cases.

A bazaar is to be held in September next in order to clear off the debt on the City and County of Cork Hospital for Diseases of Women and Children.

The Lightburn Fever Hospital, Lanarkshire, was opened on the 24th ult. Accommodation for 60 patients has been provided, at a total cost of £29 000.

We gather from the report of the Samaritan Hospital at Dublin that there has been a very much larger amount of work done by the hospital in 1895 than in 1893.

The Corporation of the City of London have sent a grant of fifty guineas in aid of Queen Charlotte's Lying-in Hospital, Marylebone, of which her Majesty the Queen is the patron.

One hundred and sixty-two in and 796 out patients were treated at the Hospital for Women, Wolverhampton, in 1895. The year's income amounted to £746, and the expenditure to £792.

The board of management of the Dewsbury and District Infirmary have decided to try the effect for twelve months of obtaining their supplies of drugs by tender from the local chemists.

The ladies' fund in connexion with the Manchester Children's Hospital has collected £1,114 for that hospital during the year 1895. Deducting £52 for expenses, a sum of £1,062 has been handed over to the hospital.

The Surbiton Cottage Hospital treated 253 patients in 1895, a number which exceeds that of any previous year. The income for the year (exclusive of £200 received in legacies and transferred to the endowment fund) was £1,056, against an expenditure of £1,049.

The committee of the Nottingham Children's Hospital, while welcoming in their report the increased usefulness of the hospital, as evidenced by the augmented number of patients, deplore the fact that the support they receive has not increased in the same proportion.

The outbreak of small-pox at Gloucester has causedthe authorities in other parts of the country to consider what means they have at their disposal for combating a similar outbreak in their midst. Leeds, Ledbury, Llandrindod, Leyton, Upton-on-Severn, East Haddingtonshire, Banff, Swindon, Middlesbrough, Stroud, Hereford, and numerous other places have all recently had the matter under discussion.

In consequence of an alteration in the arrangement of the ground floor of the Beckenham Cottage Hospital, an operating-room and two small adjoining wards have been added at a cost of £158. These wards will be used for surgical and accident cases, so that in future these classes of cases will be saved the great inconvenience and discomfort which conveyance to the general wards upstairs formerly entailed.

The Workmen's Special Fund which was inaugurated in February, 1895, in order to defray the expenses of patients at the Devizes Cottage Hospital, has not been as successful as it deserved. Although the work-people of 62 out of the 105 firms to whom collecting cards were issued became subscribers, yet in so many cases was the work of collecting only commenced and not proceeded with that at December 31st last the subscriptions amounted to but little over £19. That a sufficient amount of interest in the scheme has not yet been aroused is evidenced by the fact that out of 65 persons invited to a recent meeting only six persons attended.

### THE STUDENT OF MEDICINE.

### APPOINTMENTS.

Dr. J. B. Bowden, appointed Medical Officer for the Second District of the St. Austell Union. T. F. Devane, L.R.C.P.E., L.R.C.S.E., appointed Medical Officer to the Linen and Woollen Drapers' Institute for the Districts of Penge, Anerley, and Sydenham. A. M. Fraser, M.B., D.P.H., appointed Port Sanitary Medical Officer and Medical Officer of Health to the Borough of Portsmouth. E. L. Freer, M.R.C.S.Eng., appointed Honorary Assistant Surgeon to Birmingham Royal Orthopædic and Spinal Hospital. T. W. H. Garstang, M.A.Oxon., M.R.C.S.Eng., appointed Medical Officer of Health to the Winsford Urban District Council. C. J. Gorringe, appointed Assistant Medical Officer to the Workhouse of the Sheffield Union. J. Green, M.R.C.S.Eng., L.S.A., appointed Medical Officer of Health to the Heath Town Urban District Council. R. C. Jameson, M.B., C.M.Edin., appointed Junior Assistant Surgeon to the Huddersfield Infirmary. Dr. J. J. Johnson, appointed Medical Officer for the King's Sutton District of the Brackley Union. J. R. Kaye, M.B., C.M.Glasg., D.P.H.Camb., appointed Medical Officer of Health to the West Riding County Council. D. A. Macgregor, M.B., C.M.Edin., appointed Medical Officer of Health to the Denby and Cumberworth Urban District Council. W. L. W. Marshall, M.R.C.S.Eng., L.S.A., appointed Honorary Surgeon to the Huddersfield Infirmary. J. C. Martin, L.R.C.P.I., L.R.C.S.I., L.M., appointed Assistant Medical Officer of Donegal District Asylum, Letterkenny. W. R. Meyer, L.S.A.Lond., appointed Medical Officer for the Fourth District of the Cuckfield Union. T. Morgan, L.R.C.P.I., L.R.C.S.I., appointed Medical Officer for the Forden, Llandyssil, and Llanmerewig Districts of the Forden Union. H. E. Mortis, L.R.C.P.Lond., L.S.A., appointed Medical Officer for the Fifth District of the Oswestry Incorporation. L. Moysey, M.A., B.C.Cantab. appointed House Surgeon to the Paddington Green Children's Hospital. R. Nitch-Smith, M.R.C.S Eng., L.R.C.P.Lond., reappointed Resident House Physician to the Westminster Hospital. N. C. Ridley, M.B.Lond., F.R.C.S., L.R.C.P., R.N. (retired) appointed Honorary Ophthalmic Surgeon to the Leicester Infirmary. E. S. Robinson, M.R.C.S., L.R.C.P., reappointed Medical Officer of Health to the Stourport Urban District Council. A. L. Saunders, M.R.C.S.Eng., L.R.C.P.Lond., appointed Medical Officer for the First South-Eastern District of the Freebridge Lynn Union. W. K. Sibley, M.A., M.D., M.R.C.P., appointed Medical Examiner to the New York Life Insurance Company. T. G. Stevens, M.D.Lond., M.R.C.P., F.R.C.S., appointed Obstetric Physician to the Surrey Dispensary. Dr. G. S. R. Stretch, appointed Medical Officer for the Tudhoe District of the Durham Union. J. M. Troup, B.A.Cantab, L.S.A., appointed House Physician to the Paddington Green Children's Hospital. Dr. Waters, appointed Medical Officer for the Berriew District of the Forden Union. W. L. Woollcombe, F.R.C.S.E., M.R.C.S.Eng., L.R.C.P. Lond., appointed one of the Senior Honorary Surgeons to the South Devon and East Cornwall Hospital, Plymouth.

### VACANCIES.

The following vacancies are announced this week, the amount of salary in each case being given after the name of the institution:—

**Resident Medical Officers.**

Bedford General Infirmary and Fever Hospital.—House Surgeon; doubly qualified. Salary £100 per annum, with apartments, board, lodging, and washing. Appointment for one year, but eligible for re-election. Applications to the Secretary by June 5th.

### THE STUDENT OF MEDICINE—*continued.*

Bucks County Lunatic Asylum, Stone, near Aylesbury.—Assistant Medical Officer; unmarried. Salary £100 per annum, with board and furnished apartments in the Asylum. Applications to Wm. Crouch, Clerk to the Visiting Committee, County Hall, Aylesbury, by June 5th.

Buluwayo Hospital.—Resident House Surgeon; unmarried. Salary £400 per annum, with free board and lodging. Applications to be addressed to "The Chairman, Hospital Board, Buluwayo, Rhodesia," to reach not later than June 30th.

City of London Hospital for Diseases of the Chest, Victoria Park, E.—House Physician. Appointment for six months from July 1st. Board and residence provided, and salary at the rate of £30 per annum. Applications to the Secretary by June 11th.

Govan District Lunacy Board.—Assistant to the Medical Superintendent at the Asylum at Hawkhead, near Paisley. Salary £100, with board and apartments; unmarried, and not more than 30 years of age. Applications to Andrew Wallace, Clerk, 7, Carlton Place, Glasgow, by June 10th.

Hambledon Union.—Medical Officer for the Workhouse. Salary £70 per annum, with certain extra fees for midwifery cases and vaccination. Applications to Ferdinand Smallpiece, Clerk, Clerk's Office, Guildford, by June 2nd.

London Hospital, Whitechapel, E.—Medical Registrar. Salary £100 per annum. Assistant Physician; must be M.R.C.P.Lond. Applications to G. Q. Roberts, Secretary and House Governor, by June 11th.

London Lock Hospital, Harrow Road, W.—House Surgeon to the Female Hospital; doubly qualified. Salary £50 per annum, with board, lodging, and washing. Applications to the Secretary by May 30th.

North-Eastern Hospital for Children, Hackney Road, Shoreditch, N.E.—House Physician. Appointment for six months, at the expiration of which he will be required, if eligible, to serve as House Surgeon for six months. Salary as House Physician at the rate of £60 per annum; as a House Surgeon (the senior post) at the rate of £80 per annum. Must be doubly qualified. Junior House Physician; doubly qualified. Appointment for six months. No salary, but board and lodging (including washing) provided. Applications to the Secretary at the City Office, 27, Clement's Lane, E.C., by June 8th.

Queen Charlotte's Lying-in Hospital, Marylebone Road, N.W.—Resident Medical Officer. Appointment for four months. Salary at the rate of £60 per annum, with board and residence. Applications to the Secretary, by June 1st.

Royal Berkshire Hospital, Reading.—Assistant Medical Officer. Board, lodging, and washing provided. Honorarium of 10 guineas will be awarded if the duties are performed satisfactorily. Appointment for six months from July 1st. Applications to the Secretary before June 5th.

General Hospital, Barbadoes.—Junior Resident Surgeon. Appointment for three years. Salary £200 per annum, payable monthly, with unfurnished quarters. Passage paid. Applications to the Secretary, Barbadoes General Hospital, by July 8th.

Stamford Hill, Stoke Newington, Clapton, &c., Dispensary, 189, High Street, Stoke Newington, N.—Junior Resident Medical Officer. Salary at the rate of £50 per annum during the first quarter, afterwards at the rate of £75 per annum, with board and lodging. Applications to the Senior Resident Medical Officer by June 3rd.

Sussex County Hospital, Brighton.—House Physician, doubly qualified, unmarried, and under 30 years of age. Salary commencing at £50 per annum, with board and residence in the hospital, and washing. Applications to the Secretary by June 3rd.

#### Non-Resident Medical Officers.

Borough of Chesterfield.—Medical Officer of Health. Appointment for one year. Salary £300 per annum. Applications, endorsed "Medical Officer," to be sent to Jno. Middleton, Town Clerk, by June 1st.

Charing Cross Hospital.—Surgical Registrar. Salary £40 per annum. Applications to Arthur E. Reade, Secretary, by June 8th.

Manchester Clinical Hospital for Women and Children, Park Place, Cheetham Hill Road.—Honorary Assistant Physician. The selected candidate will not be allowed to hold any other hospital appointments. Applications to Mr. Hubert Teague, Secretary, 38, Barton Arcade, Manchester, by June 6th.

Middlesex Hospital, W.—Assistant Surgeon. Applications to F. Clare Melhado, Secretary Superintendent, by June 8th.

Paddington Green Children's Hospital, London, W.—Physician to Out-patients, and Surgeon to Out-patients. Applications to the Secretary by May 30th.

Queen's Jubilee Hospital, Earl's Court, S.W.—Aurist and Laryngoscopist and House Surgeon. Appointment for the latter for six months; honorarium, £100 per annum and rooms. Applications to the Secretary for the former appointments to reach by May 30th.

Royal College of Surgeons of England.—Hunterian Professors, Erasmus Wilson Lecturer, and Arris and Gale Lecturer for the ensuing years. Applications to Edward Trimmer, Secretary, by June 2nd.

Royal Eye Hospital, Southwark.—Surgeons and Assistant Surgeons and Clinical Assistants. Applications to the Secretary at the hospital by June 8th.

University Court of St. Andrews.—Lecturers on (1) Anatomy; salary £300 per annum, with class fees. (2) Materia Medica; salary, £200, with class fees. (3) History; salary, £200, with class fees. Applications to Mr. Stuart Grace, Secretary to the University Court, by June 1st.

